# Disease Compass– a navigation system for disease knowledge based on ontology and linked data techniques

**DOI:** 10.1186/s13326-017-0132-2

**Published:** 2017-06-19

**Authors:** Kouji Kozaki, Yuki Yamagata, Riichiro Mizoguchi, Takeshi Imai, Kazuhiko Ohe

**Affiliations:** 1 The Institute of Scientific and Industrial Research, Osaka University, 8-1 Mihogaoka, Ibaraki, Osaka 567-0047 Japan; 2 National Institute of Biomedical Innovation, Health and Nutrition, 7-6-8, Saito-Asagi, Ibaraki, Osaka 567-0085 Japan; 3 Research Center for Service Science, Japan Advanced Institute of Science and Technology, 1-1 Asahidai, Nomi, Ishikawa 923-1292 Japan; 4 Graduate School of Medicine, The University of Tokyo, 7-3-1, Hongo, Bunkyo-ku, Tokyo, 113-0033 Japan

**Keywords:** Disease ontology, Definition of diseases, River flow model of disease, Linked data, Navigation system

## Abstract

**Background:**

Medical ontologies are expected to contribute to the effective use of medical information resources that store considerable amount of data. In this study, we focused on disease ontology because the complicated mechanisms of diseases are related to concepts across various medical domains. The authors developed a River Flow Model (RFM) of diseases, which captures diseases as the causal chains of abnormal states. It represents causes of diseases, disease progression, and downstream consequences of diseases, which is compliant with the intuition of medical experts. In this paper, we discuss a fact repository for causal chains of disease based on the disease ontology. It could be a valuable knowledge base for advanced medical information systems.

**Methods:**

We developed the fact repository for causal chains of diseases based on our disease ontology and abnormality ontology. This section summarizes these two ontologies. It is developed as linked data so that information scientists can access it using SPARQL queries through an Resource Description Framework (RDF) model for causal chain of diseases.

**Results:**

We designed the RDF model as an implementation of the RFM for the fact repository based on the ontological definitions of the RFM. 1554 diseases and 7080 abnormal states in six major clinical areas, which are extracted from the disease ontology, are published as linked data (RDF) with SPARQL endpoint (accessible API). Furthermore, the authors developed *Disease Compass*, a navigation system for disease knowledge. *Disease Compass* can browse the causal chains of a disease and obtain related information, including abnormal states, through two web services that provide general information from linked data, such as DBpedia, and 3D anatomical images.

**Conclusions:**

*Disease Compass* can provide a complete picture of disease-associated processes in such a way that fits with a clinician’s understanding of diseases. Therefore, it supports user exploration of disease knowledge with access to pertinent information from a variety of sources.

**Electronic supplementary material:**

The online version of this article (doi:10.1186/s13326-017-0132-2) contains supplementary material, which is available to authorized users.

## Background

Recently, medical information resources that store considerable amount of data have become available. Semantic technologies are expected to contribute to the effective use of such information resources, and medical ontologies such as the Systematized Nomenclature of Medicine-Clinical Terms (SNOMED-CT, http://www.nlm.nih.gov/research/umls/Snomed/snomed_main.html main.html) and the Ontology of General Medical Sciences (OGMS) [1] have been developed to realize sophisticated medical information systems. Although medical ontologies consist of various domains, such as diseases, anatomies, drugs, and clinical information, disease is a particularly important concept because diseases have complicated mechanisms that are deeply related to concepts across many medical domains. Therefore, in this study, we have focused on a disease ontology.

Although disease ontologies such as the Human Disease Ontology (DOID) [2] and the Infectious Disease Ontology (IDO) [3] exist, they primarily focus on the ontological definition of a disease with related properties, i.e., static aspects of diseases are the main concern. While the OGMS provides an ontological representation model of disease disposition, it does not capture a complete picture of disease-associated processes.

In contrast, we proposed a definition of a disease that captures the causal chain of the abnormal states in a computational model known as the River Flow Model (RFM) of diseases [4, 5]. Our disease ontology consists of rich information about these causal chains, which provides domain-specific knowledge about diseases and answers questions such as “What disorder/abnormal state causes a disease?” or “How might the disease advance, and what symptoms may appear?” Consequently, we believe that the ontology could be a valuable knowledge base for advanced medical information systems.

In this paper, we discuss a fact repository for causal chains of disease based on our disease ontology. It is developed as linked data so that information scientists can access it using friendly SPARQL queries through an RDF model for causal chain of diseases. We designed the RDF model as an implementation of the RFM for the fact repository while ontological definitions of the RFM are discussed in our previous work [4, 5]. It provides known knowledge about mechanism of disease to support education for novice clinicians, differential diagnosis, decision making for medical treatment and so on.

In this paper, we also describe *Disease Compass*, a navigation system for disease knowledge based on the RDF model of our disease ontology. The system has two special features. First, users can browse disease knowledge according to the causal chains of diseases defined in the disease ontology. Second, users can obtain related information about the selected disease from linked data sources. Thus, *Disease Compass* helps users make sense of disease knowledge from various relevant sources.

The remainder of this paper is organized as follows. The methods used to develop our disease ontology and navigation system are introduced in Methods section. In Results section, we describe the disease ontology in detail and discuss how it can be published as linked data and *Disease Compass*. In Discussion section, we discuss our contributions from the perspective of the ontological definition of disease and medical information systems. Conclusions and suggestions for future work are presented in Conclusions section.

This paper is an extended version of the conference paper presented in ICBO2015. It is mainly added the following 3 topics; 1) details of integration of biomedical abnormal states discussed in Integration of biomedical abnormal states section, 2) details of development of Disease Compass, especially about its navigation function for general causal chains, and 3) Some concrete examples of how the system is used with discussions.

## Methods

We developed the fact repository for causal chains of diseases based on our disease ontology [4, 5] and abnormality ontology [6, 7]. This section summarizes these two ontologies.

### Definition of a Disease

#### Basic definition

Based on the RFM, we define a disease as follows [4].

#### Definition 1

A disease is a dependent continuant constituted of one or more causal chains of clinical disorders (abnormal states) that appear in a human body and is initiated by at least one disorder.

In this definition, by clinical disorders (abnormal states), we mean states in a human body which consists of causal chains of a disease. They are associated with dysfunctional or otherwise pathological functioning in organisms while they may not be abnormal in some cases. They are defined in our Abnormality Ontology [7] as mentioned in Abnormality Ontology section.

Then, what is a causal chain of disorders? Although it looks like a process, it is a dependent continuant. It is possible to compare a causal chain of disorders to a waterfall, river flow, or a forest fire. Here, we show how a disease is a dependent continuant rather than a process. The following is an informal account of our view. This topic is extensively discussed with ontological definitions of related concepts in the literature [5].

A causal chain is composed of one or more pairs of entities, such as a causal event and an effect event, in which the latter has been caused by the former. In the case of multiple-pair chains, the effect becomes another cause that causes another effect. What makes clinical causal chains special is that causal entities are usually still active when the effect entity has been caused. Therefore, the two entities overlap in temporal space. In the case of continuant entities, by “overlap” we mean that the intervals of active states of neighboring continuants overlap, i.e., the causal continuant maintains its state when the effect state has been caused.

Let us examine how well a flowing river matches a causal chain of a disease. The river itself enacts branching, changing shape, extending, diminishing, etc. A river could be created when a lake overflows, e.g., after a heavy rainstorm. Initially, the flow is minimal and, potentially, temporary. Here, overflow from a lake would correspond to an etiological disorder in a clinical causal chain. If the initial flow increases, the water extends in length and is recognized as a river. After emerging as a river (as a disease), it extends further to another lake or to the sea. While extending, it branches (branching can be the appearance of another disorder). Eventually, the river may dry up due to climate change (cure). Thus, the life of a river corresponds well to the life of a disease. Note that a river is defined as an enactor of those processes, and Definition 1 suggests that a disease is defined as an enactor of its manifestation process. Thus, in concordance with OGMS, both a river and a disease are continuants; however, a river is an *independent* continuant and a disease (causal chain) is a *dependent* continuant that depends on a bearer, i.e., an organism.

This informal observation is supported by ontological accounts of processes and objects [8]. Although we omit details because of space limitations, we present the analogy to support the definition of a disease as a dependent entity of a new type that differs from both a disposition and a process.

#### Granularity

We do not specify any particular granularity of disorder and causal chains because we believe granularity should be determined flexibly according to the necessity of description of each disease. That is, we define diseases based on the most agreeable medical knowledge at this time because the current medical knowledge changes as time goes. However, with regard to the original cause, we should trace the causal chain back to the cell level rather than to the genome level. When we define diseases generally, granularity is not an issue; however, it matters when we define a particular disease in the ontology.

In addition, we do not impose specific time resolution on the causal processes, so that, if necessary, we can include rapid processes, such as fractures. After receiving a strong external pressure, a bone undergoes a very quick destruction process resulting in fracture. The causal process can be captured by much finer time resolution than those involved in ordinary pathological processes captured at the clinical level. Fractures can be handled by the disease model discussed in the next section.

#### Related work

Here we compare the definitions of diseases in the OGMS and RFM.

1. Dispositions are introduced in the course of disease development in the human body. A disposition is a potentiality. In the current OGMS, realization of this potentiality takes the form of chains of physical/physiological changes. Thus, disease and disease course are distinguished, that is, the former is dependent continuant and the latter a process. We believe this use of “disease” is counterintuitive to clinicians; thus, we propose a disease definition that allows the disease to be understood as a causal chain of abnormal states.
2. Consider how a particular disease is identified. For example, when explaining diabetes, OGMS refers to an “elevated level of glucose in the blood.” However, it provides an insufficient account of why the explanation of diabetes must include “elevated level of glucose.” What role does this elevated level play in diabetes? Why must “elevated level of glucose in the blood” be included for diabetes but nothing else? It must be something specific to the disease of interest, i.e., each realization of the disease must involve an entity of this sort. For OGMS, emphasis is placed on the disposition and the disorder (a certain disordered body part) in which this disposition inheres. We believe that the reference to elevated glucose level suggests a need for an additional entity, which is included in our disease model. Thus, we introduce the notion of *core causal chain,* which roughly corresponds to so-called *main pathological/etiological condition(s).*

We know that defining such an entity type, i.e., causal chains, discussed in (2) is difficult because such causal chains are not always definite for each disease because they vary from one patient to another. Hence, OGMS’ use of disposition is a mere potentiality. In the case of latent diabetes, for example, there is no elevated level of glucose in the blood, although there is a disposition thereto. Accordingly, for latent diabetes, we follow OGMS in recognizing the need for something other than just “elevated level of glucose in the blood.” However, we think that something more is required–something that is essential for each particular disease. In the case of diabetes, this would be the *deficiency of functioning of insulin*, because this must have occurred for all patients who suffer from diabetes. To address this issue, we draw on OGMS’ notion of homeostasis and introduce the term “disturbance of homeostasis” to explain what we consider as the essential core of each disease. Disturbance of homeostasis can be caused through the concretization of a disposition, or it can be caused by some outside trigger, e.g., an injury.

We agree with OGMS in that a disease is a dependent continuant, and its definition is expected to address the following conditions: (1) the existence of its pre-clinical manifestation, (2) the fact that it can cause another disease, and (3) variation in the disease course from patient to patient [1]. We have attempted to find a disease definition that satisfies these conditions using an RFM [4].

### Abnormality Ontology

#### Three-Layer Ontological Model of Abnormal States

Here we discuss the abnormal states used in our disease ontology to define diseases. The reliability and utility of the disease definitions are considerably dependent on the quality of the abnormal states. To develop abnormal states consistently, we have developed an abnormality ontology [6, 7] with a three-layer structure:

- Level 1: Generic abnormal states
  Level 1 defines very fundamental (or generic) concepts, which do not depend on any structural entity, i.e., object independent states. They are commonly found in several objects, and can be usable in several domains besides medicine, such as machinery, materials, and aviation.
- Level 2: Object-dependent abnormal states
  Level 2 has been developed by identifying the target object and specializing generic abnormal states at Level 1 with consistency. The top level concepts at Level 2 are dependent on generic structures, such as “wall-type structure,” “tubular structure,” and “bursiform structure,” which are common and are used in several domains.
- Level 3: Specific context-dependent abnormal states
  Level 3 consists of context-dependent abnormal states, which refer to the Level 2 abnormal states to define them, and are specialized into specific disease-dependent ones.

Level 1 defines very fundamental and generic concepts, e.g., “small in area,” “hypofunction,” etc., which are commonly used in clinical medicine and other domains. Therefore, they do not have a target entity which has the abnormal state. Level 2 concepts are dependent on objects. In the lower level of the tree, concepts are designed to represent abnormalities at specific human organ/tissue/cell levels. For example, by specifying “small in area” at Level 1, “tube narrowing,” where the cross-sectional area of a tubular structure has become narrowed, is defined at Level 2. This is further specified in the definitions “vascular stenosis” (blood vessel-dependent), “arterial stenosis,” “coronary artery stenosis” (coronary artery-dependent), and so on. Level 3 concepts are captured as specific disease-dependent (context-dependent) abnormal states. For example, “coronary artery stenosis” at Level 2 is defined as a constituent of ischemic heart disease at Level 3. In the proposed ontological approach, common concepts can be kept distinct from specific concepts and can be defined appropriately according to their context.

#### Representation of Abnormal State

In medicine, abnormal states are interpreted from the diverse perspectives of specialists, such as clinicians, pathologists, biologists, and geneticists, and correspondingly a variety of representations of abnormal states are used. Therefore, we have classified abnormal states into three categories: a property (e.g., hypertension), a qualitative representation (e.g., blood pressure is high), and a quantitative representation (e.g., blood pressure 180 mm Hg). Their interdependence is formulated in a Property-Attribute interoperable representation framework for abnormal states we proposed in previous work [6, 7].

We capture all abnormal states as properties represented by a tuple: <Property (P), Property Value (Vp)>, e.g., <stenosis, true>. Apparently, any state requires temporal specification as well as its bearer. For simplicity, these temporal indexes are omitted. However, the bearer is specified to represent the fact that it is in a state, as discussed below. We specify the property by decomposing it into a tuple: <Attribute (A), Attribute Value (V)>. The Attribute Value can be either a Qualitative Value (Vql) or a Quantitative Value (Vqt). For example, “arterial stenosis” is decomposed into < cross-sectional area (A), small (Vql) > as a qualitative representation, or < cross-sectional area (A), 5 mm^2^ (Vqt) > as a quantitative representation.

Then, we introduce “Object” to identify the target object, and we represent an abnormal state as a triple: <Object (O), Attribute (A), Value (V)>. This is the basic form of abnormalities in our representation model. In addition, we introduce “Sub-Object” (SO) as an advanced representation of what will be focused on. For example, in the case of “hyperglycemia,” since the glucose concentration (A) means the ratio of the focused object (SO) relative to the whole mixture (O), the representation of “hyperglycemia” is a quadruple, <blood (O), glucose (SO), concentration (A), high (V)>. In an advanced representation, “colonic polyposis” is described as < colon (O), polyp (SO), number (A), many (V) > .

Our model can deal with both clinical test data and abnormal states in disease definitions. Clinical test data can be represented in the form < Object (O), Attribute (A), Quantitative Value (Vqt) > (OAVqt), which can be converted into a property representation form < Object (O), Property (A), Property Value (Vp) > (OPVp) via a qualitative representation form. For example, in terms of the state of hypertension, our model ensures interoperability among the forms < blood (O), pressure (A), 180 mmHg (Vqt)>, <blood pressure, high>, and < hypertension, true>. Therefore, our model realizes interoperability between test data and abnormal states in the definition of diseases. The ontological foundation for the concepts discussed thus far is given by an upper ontology, i.e., YAMATO [9].

### Ontology Editing

We used the Hozo (http://www.hozo.jp) [10] ontology editing tool. Hozo is based on an ontological theory of role [11] and has a sophisticated graphical user interface. Although Hozo uses a proprietary ontology format based on XML, it can export ontologies in Web Ontology Language (OWL) [12]. An API for use the Hozo format is also available at the website of Hozo. We also developed a graphical tool that allows clinicians to edit a disease definition intuitively without prior knowledge of ontology construction. The tool can export disease definitions in the Hozo ontology format.

### System Development based on Linked Data Technology

There are several approaches for system development based on ontologies. A typical approach is to use APIs for ontology processing. Because our disease ontology is constructed using Hozo, we can develop application systems using APIs for Hozo ontologies. We can also use the OWL API because Hozo has an OWL export function. However, linked data technology is particularly efficient for developing applications across multiple datasets on the web. Therefore, we adopted an alternative approach to publish the disease ontology as linked data so that it can be used to develop an application system easily.

At the same time, the schema of RFM is published at http://rfm.hozo.jp/ the Hozo format and the OWL format. If the users are familiar with OWL, they can use the OWL version in spite of our disease ontology is currently published only as Linked Data through its SPARQL endpoint (see Disease ontology as linked data section).

## Results

### Computational Model of Diseases

#### Core causal chain of a disease

Based on the disease ontology based on RFM, we build a computational model of diseases to make it easier to define particular diseases. In the following, we divide diseases into (Type 1) those whose etiological and pathological processes are well understood and (Type 2) other diseases.

Type 1 diseases are identified by their inherent etiological/pathological process(es). Type 2 diseases include so-called syndromes and are typically represented in terms of *criteria for diagnosis*. We deal with Type 1 diseases first. Note that every Type 1 disease should have a clue to identify the disease. In other words, we should be able to find something similar to the so-called *main pathological/etiological condition(s)* that theoretically characterize(s) the disease. As stated above, this is what OGMS should include. We know that Type 2 diseases necessarily employ *criteria for diagnosis* to identify them because of a lack of knowledge about their etiological/pathological processes. However, this does not mean Type 2 disease is excluded from our disease model as discussed below, which we share with OGMS.

We also need a formulation to organize diseases in an *is-a* hierarchy in a disease model. According to our definition, a disease can be represented as a directed graph consisting of disorders as nodes and causal links. An *is-a* relation between diseases using an inclusion relationship between causal chains can be described as follows.

#### Definition 2: *Is-a* relation between diseases

Disease A is a super class of disease B if all causal chains at the class level of disease A are included in those of disease B. The inclusion of nodes (disorders) is determined by considering an *is-a* relation between the nodes, as well as the sameness of nodes.

#### Definition 3: Core causal chain of a disease

The causal chain of a disease included in the chains of all its subclass diseases is called the core causal chain.

**Fig. 1:**
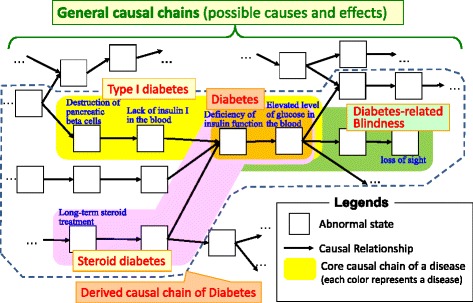
Types of diabetes composed of causal chains

Definition 3 helps us capture the necessary and sufficient conditions of a particular disease systematically, which roughly corresponds to the so-called “main pathological/etiological conditions.” Figure 1 shows one of the main types of diabetes constituted by corresponding types of causal chains. The most generic type in this example is *(non-latent) diabetes*, which is constituted by the following chain:

- *deficiency of insulin → elevated level of glucose in the blood.*

The next lower subclasses include *Type-I diabetes*, which is constituted by:

- *destruction of pancreatic beta cells → lack of insulin I in the blood → deficiency of insulin → elevated level of glucose in the blood,*

and *steroid diabetes*, which is constituted by:

- *long-term steroid treatment → … → deficiency of insulin → elevated level of glucose in the blood.*

If a doctor wanted a hierarchy that represents diabetes-caused blindness, it would be:

- *deficiency of insulin → elevated level of glucose in the blood → … → loss of sight.*

Although we explain the disease model using Type 1 diseases, the model is also applicable to Type 2 diseases because of the flexibility of granularity and degree of being “well-understood.” These two kinds of flexibility can be exploited according to each disease. In the case of Type 2 diseases, we could employ an “unknown” causal node linking to just a few of the symptoms that are typically observed in the syndrome under consideration. Note that this model can capture a seemingly isolated symptom by combining it with an unknown cause to form a causal network. It also captures diseases with multiple causal chains.

In addition, the proposed model can distinguish, for example, between diabetes with blindness and diabetes-driven blindness by specifying the core causal chain of focus. In summary, the disease model yielded by the proposed definition of disease (Definition 1, Disease ontology as linked data section) covers a wide range of diseases. In fact, we have constructed models of 6051 diseases from 12 different divisions in our ontology, which shows the expressive power of the proposed disease model.

#### Types of causal chains in disease definitions

In theory, when we define a disease, we can consider three types of causal chains that appear in the definition of disease.

**General Causal Chains** are all possible causal chains of (abnormal) states in a human body. They can be referred to by any disease definition.

The **Core Causal Chain** is a causal chain that appears in all patients that have the disease.

**Derived Causal Chains** are causal chains that are obtained by tracing general disease chains upstream or downstream from the core causal chain. Upstream chains imply possible causes of the disease, whereas downstream chains imply possible symptoms in a patient suffering from the disease.

The core causal chain represents a stable definition of the disease. That is, it defines only such causal chains that appear in all patients. On the other hand, the general causal chains and derived causal chains are not part of the definition but possible causal chains which might not appear in some patients. That is, the general causal chains and derived causal chains represents possibilities how causal chains could be extended.

Figure 1 shows how the main types of diabetes are composed of corresponding types of causal chains. The figure shows that diabetes subtypes are defined by extending the disease’s core causal chain according to its derived causal chains (upstream or downstream).

Note that it is obviously difficult to define all general causal chains in advance, because it is impossible to know all possible states in the human body and their causal relationships. To overcome this difficulty, we define general causal chains by generalizing the core/derived causal chains of every disease defined by clinicians using a bottom-up approach. We asked clinicians to define only core causal chains and typical derived causal chains of each disease according to their existing knowledge and information that can be found in textbooks. General causal chains are then defined by generalizing these definitions.

#### The scope of the model for disease

In this section, we discuss the scope of the proposed model of diseases. This paper focuses on how to capture diseases and the implementation of diseases as causal chains based on the RFM. As mentioned in Definition of a Disease section, we assume that ontological definitions of causal chains, diseases, and abnormalities (disorders) etc. are out of the scope of this paper since they are discussed in our previous papers [4, 5].

At first, we have to distinguish the following two problems when we represent a disease.

1. How we can represent causal chains of diseases as a computational model
2. How far the current medical knowledge reveals the causal mechanism of diseases?

This paper focuses on the former and the latter problem is out of scope. That is, we discuss how the model capture the current medical knowledge about diseases based on the RFM.

We here classify difficulties for defining diseases as causal chains into the following two types;

1. Problems of grain size to describe causal chains.
2. How far we can follow causes of diseases.

For the former, we use the Abnormality Ontology we proposed in our previous work [7]. It supports multi levels representations of abnormal states according to their grain size.

For the latter, we carefully investigated how we should represent causal chains of diseases through discussions with medical experts including clinicians. As the result, we introduced a flexible representation model which we can describe causal chains according to a range of given knowledge even if some causes of the disease are unknown. When causes of an abnormal state is unknown, we represent it “unknown node” in the causal chain. It means that the model can represent causal chains according to how far we can follow causes of diseases. On the other hand, when we know that an abnormal state has several causes, we can also represent it using multiple chains. Please note that multiple chains can be represented using AND/OR graphs which is a simple and basic knowledge representation. Although representations of AND/OR in OWL are somewhat complicated, this is why we publish causal chains of diseases as not OWL but simple RDF graph as discussed in Disease ontology as linked data section.

#### Implementing the Disease Ontology

We developed the disease ontology using Hozo. Although Hozo is based on an ontological theory of roles and has its own ontology representation model, we show an OWL representation of the ontology to aid understandability. Note that we use a simplified OWL representation of the disease ontology to provide an overview; however, it does not support the full semantics of Hozo. The detailed semantics of Hozo are discussed in the literature [12].

**Fig. 2:**
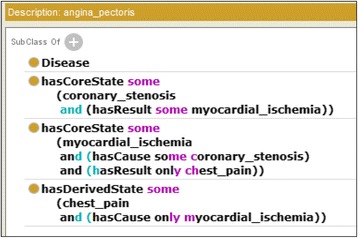
Class definition of *angina pectoris* in OWL

**Fig. 3:**
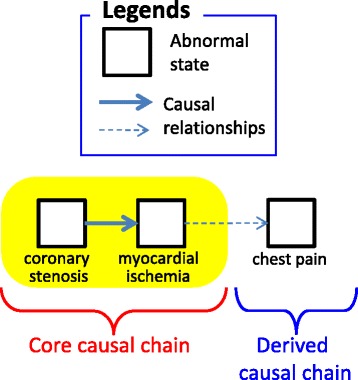
Causal chain of *angina pectoris*

**Fig. 4:**
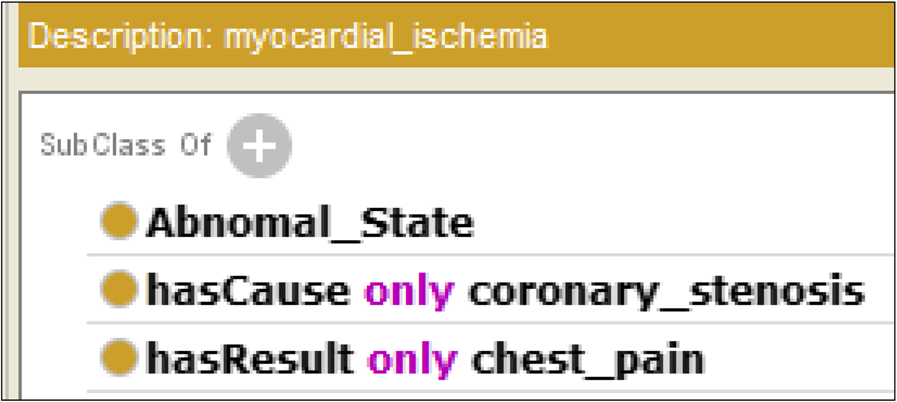
Class definition of myocardial ischemia in OWL

Figure 2 shows an OWL representation of *angina pectoris*, whose causal chain is shown in Fig. 3. Abnormal states that appear in the disease are listed using the *owl:Restriction* properties on the *hasCoreState*/*hasDerivesState* properties. The former represents abnormal states in its core causal chain, and the latter represents those in its derived causal chain as defined by a clinician. The causal relationships among them are represented by *hasCause*/*hasResult* properties. If the probability of the causal relationship is high, *hasProbableCause*/*hasProbableResult* properties are used instead. However, how this probability is determined is beyond the scope of this paper. Causal chains (states and causal relationships among them) in core causal chains are necessary (Definition 3); therefore, the *owl:someValuesFrom* properties are used. On the other hand, because causal chains in derived causal chains are possible, *owl:allValuesFrom* properties are used to represent the possible causes/results. If there are more than two possible causes/results, *owl:unionOf* is used to list them. The definitions of diseases refer to the definitions of abnormal states, which represent the possible causes and results, as shown in Fig. 4. General disease chains are represented as an aggregation of the definitions of abnormal states.

Our disease ontology has been developed in collaboration with clinicians from 13 fields, such as cardiology, neurosurgery, and allergology. As of May 11, 2013, it contained approximately 6302 disease concepts and 21,669 disorder (abnormal state) concepts with the causal relationships that exist among them. We keep to revise them mainly focusing on major diseases.

### Disease ontology as linked data

#### Basic policy to publish the disease ontologies as linked data

The standard format for linked data is RDF; thus, one might consider it easy to publish ontologies in RDF formats using OWL or RDF(S) as linked data. However, ontology languages, such as OWL, are primarily designed for class descriptions, and there is an assumption that the language will be used for reasoning based on logic. In contrast, for linked data, finding and tracing connections between instances is the primary task. Therefore, for convenience or efficiency, OWL and RDF(S) are not always appropriate for linked data because of their complicated graph structures.

**Fig. 5:**
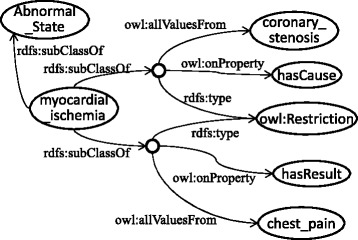
RDF graph of a general disease chain in OWL

For example, when we obtain a general disease chain, which is probably caused by *myocardial_ischemia*, we must repeat SPARQL queries to obtain RDF graphs, which include blank nodes, such as those shown in Fig. 5. Furthermore, when we obtain the definitions of a disease, we must perform more complicated queries to obtain graphs that correspond to OWL descriptions, such as those shown in Fig. 3, with restrictions inherited from the disease’s super classes. These queries and graph patterns are intuitively very different from the disease chains we want to produce. It is due to restrictions from the super classes.

This is problematic, especially when the conceptual structures of the ontology are intended for use as a knowledge base with rich semantics. Consequently, we have designed an RDF data model in order to publish our disease ontology as linked data [13].

#### RDF model for causal chains of diseases

Once the disease ontology was constructed, information about the causal chains of diseases was extracted and converted into RDF format as linked data. We call this dataset *Disease Chain-LD*, and it consists of diseases, abnormal states, and the relationships among them. Abnormal states are represented by instances of the *Abnormal_State* type, and the causal relationships between them are represented by *hasCause* and *hasResult*, which are inverse properties. The abnormal states connected by these properties are a possible cause/result; therefore, general disease chains can be obtained by collecting all abnormal states according to these connections.

Diseases are represented by instances of *Disease* type. Abnormal states that constitute a core causal chain and a derived causal chain of a disease are represented by *hasCoreState* and *hasDerivedState* properties, respectively. *Is-a (sub-class-of)* relationships between diseases and abnormal states are represented by *subDiseaseOf/subStateOf* properties rather than *rdfs:subClassOf* because the diseases and abnormal states are represented as RDF resources, whereas *rdfs:subClassOf* is a property between *rdfs:Classes.*

**Fig. 6:**
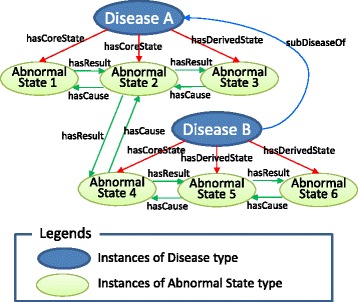
RDF representation of disease

**Fig. 7:**
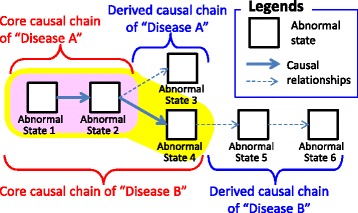
Causal chains of diseases shown in Fig. 2

Figure 6 shows an example of an RDF representation of diseases. It represents *disease A* and its sub-disease *disease B*, whose causal chains are shown in Fig. 7. Note that the causal chains consist of abnormal states and the causal relationships among them. Therefore, when we obtain a disease’s core causal chain or derived causal chain, we must obtain both the abnormal states connected to the disease by *hasCoreState/hasDerivedState* properties and the causal relationships among them. Although causal relationships are described without determining whether they are included in the causal chains of certain diseases, we can identify the difference of abnormal states which diseases include by assessing whether the abnormal states at both ends of *hasCause/hasResult* properties are connected to the same disease by *hasCoreState/hasDerivedState* properties. Furthermore, for a disease that has a super disease, such as disease B in Fig. 7, in addition to obtaining the causal chain directly connected with the disease, we must also obtain the causal chains of the super disease, and these chains must be aggregated.

We have published the disease ontology as linked data based on our RDF model. It includes the definitions of 1.554 diseases and 7080 abnormal states in six major clinical areas, which were extracted from the disease ontology on June 24, 2014. At this point, the dataset contains 61,473 triples. Although the disease ontology includes the definitions of diseases in 13 clinical areas, we have published only those parts that were well reviewed by clinicians. A SPARQL endpoint to access the disease ontology is published at http://lodc.med-ontology.jp/. The users can find concrete examples of causal chains of diseases in RDF through this endpoint using SPARQL queries as discussed in the next section. Furthermore, we also provide user friendly navigation system for causal chains of disease as discussed in Development of *Disease Compass* section.

#### Example Queries

The processing is not complicated; it requires only simple procedural reasoning. We can obtain the causal chains that define a disease through several SPARQL queries to the dataset [13].

**Fig. 8:**
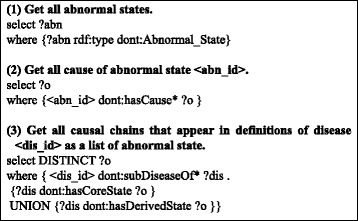
Example queries. Here, “dont:” represents a prefix of the Disease Chain-LD and < abn_id > and < dis_id > represent the id of a selected abnormal state and disease, respectively

Figure 8 shows example queries to obtain an abnormal state in the dataset. Because all abnormal states are defined as individual resources *of* the *Abnormal_State* type, we can obtain them using the query shown in (a1). When we want to obtain the causes/result of a selected abnormal state, we can follow the *hasCause/hasResult* properties. For example, (a2) is a query to obtain all causes of the selected abnormal state. Furthermore, we can obtain a general disease chain that includes the abnormal state using the query shown in (a3). This query means to follow all of the *hasCause/hasResult* properties recursively from the selected abnormal state.

**Fig. 9:**
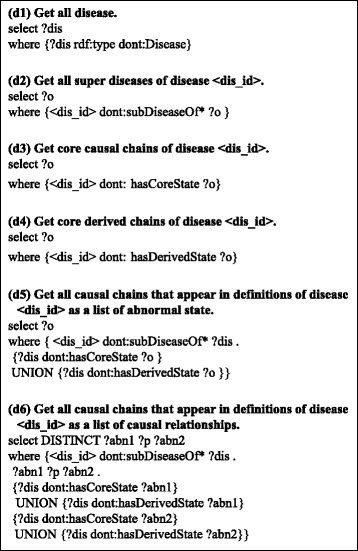
Example queries to obtain definitions of diseases. In this figure “dont:” represents a prefix of the Disease Chain LOD, and < dis_id > represents the id of a selected disease

On the other hand, Fig. 9 shows example queries to obtain the definitions of diseases. We can obtain all diseases in the dataset using query (d1), which is similar to query (a1). When we want to obtain all super diseases (super class) of a selected disease, we can use the query show in (d2). To obtain the core causal chains or derived causal chains of a selected disease, we can use query (d3) or (d4), respectively. By combining queries (d2), (d3), and (d4), we can obtain all causal chains that appear in the definitions of the disease using the query shown in (d5). Furthermore, when we want to obtain a list of causal relationships that appear in the causal chain of the definition of the diseases rather than a list of abnormal states, we can use the query shown in (d6). This query finds all properties among all abnormal states that appear in the definition of the selected disease.

We believe that all of the above queries are easy to understand and intuitive for many people. This is a significant advantage of our RDF model compared to our original OWL disease ontology.

#### Integration of biomedical abnormal states

##### Mapping to Other Resources

As illustrated in the previous section, our ontology provides three levels of abnormal states, from generic to disease-specific.

**Fig. 10:**
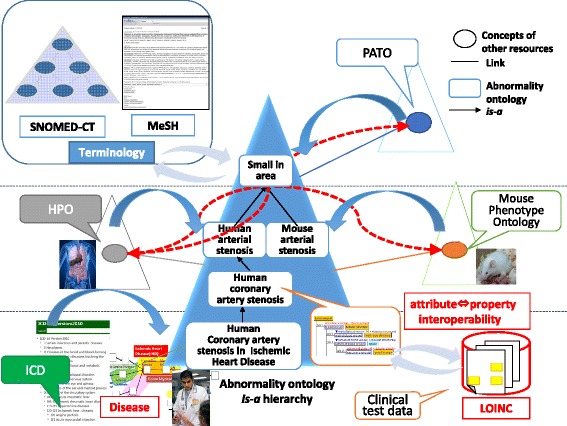
Integration of abnormal states in biomedicine

Level 1 in our ontology defines generic concepts that correspond to the PATO concepts [14], and those concepts can be mapped to related PATO concepts (Fig. 10). The lower Level 2 concepts are human anatomical structure-dependent abnormal states that correspond to Human Phenotype Ontology (HPO) concepts [15]. By creating links between Level 2 concepts and HPO concepts, it becomes possible to navigate from the HPO concepts to the upper generic PATO concepts. Level 3 provides disease-specific abnormal states, such as “myocardial ischemia in ischemic heart disease” and “chest pain in angina pectoris.” In the revised version 11 of the International Classification of Diseases (ICD), diseases contain “causal properties” information [16]; therefore, we plan to map our Level 3 concepts to the corresponding ICD concepts. Level 3 abnormal states are described in the causal chains of diseases [4]. By mapping our disease concepts of disease ontology to the ICD, ICD users can understand the causal relationships of the abnormal states in diseases. Our ontology also allows users to navigate related concepts in other resources, such as HPO and PATO.

Mapping our ontology to other resources in order to integrate various data related to abnormalities will also provide benefits to the users of other resources. First, one can find concepts from generic to specialized terms easily by referring to the single *is-a* tree in our abnormality ontology. For example, although HPO does not consider consistent *is-a* relationships in terms of “stenosis,” by referring to “arterial stenosis” at Level 2 in our ontology through mapping, HPO users can obtain the following *is-a* relationships: “arterial stenosis *is-a* vascular stenosis *is-a* narrowing tube *is-a* small in area.”^1^ Since “small area” is linked to a PATO concept, via our ontology, users might find orthologous concepts of other species. In particular, human phenotypes can be linked to the phenotypes of model organisms, e.g., mouse and rat, if the set composed of Attribute (A) and Value (V) are identical and the Object (O) has structural similarity. PATO2YAMATO attempts to integrate phenotype descriptions residing in differently structured comparison contexts [17]. By applying PATO2YAMATO, mapping concepts across species and integrating knowledge from various species may be possible.

We also plan to link the components of the < Object (O), Attribute (A), Value (V) > representation of abnormal states to other resources. For example, we suppose that Object (O) can be linked to concepts in the Foundational Model of Anatomy (FMA) ontology [18]. By mapping medical terminologies, such as SNOMED-CT[19] and MeSH terms [20], it will be possible to retrieve biomedical articles related to abnormal states or diseases. It will be also useful for the users of these terms to understand how their research subjects are involved in various abnormal states in the human body relative to diseases.

##### Trial integration

In order to assess the feasibility of the above approach, we conducted a trial integration of some examples taken from the three-level ontology of abnormal states, the disease ontology, and some typical external resources, in which we used 386 abnormal states consisting of 279 abnormal states that are super classes of 107 states that are the bottom level classes appearing in 12 typical diseases from three medical specializations, i.e., cardiovascular medicine, neurology, and gastroenterology. Mapping an abnormal state defined in the abnormal state ontology to related concepts in external resources was performed manually after detecting candidates using a perfect string match algorithm.

Table 1 shows the mapping results for PATO, HPO, MeSH, and SNOMED-CT for each level of abnormal states. As can be seen in the table, 52 abnormal states in PATO are mapped to those among 134 states at Level 1 of the abnormal ontology, none in HPO, two in MeSH, and none in SNOMED-CT. No abnormal state is found among the 107 states at Level 3 in the four external sources because they are disease-specific abnormal states. It is interesting to find that abnormal states at Level 2 have more corresponding states in external resources than those at other levels. This is because those at Level 1 are too abstract and those at Level 3 are too specific. Some examples of mapping results are shown in Table 2.

**Table 1:** Mapping between abnormal state ontology and external resources. Some examples of mapping results are shown in Table 2

Concepts	Our Ontology	PATO	HPO	MeSH	SNOMED–CT
Level 1	134	52	0	2	0
Level 2	145	2	27	28	17
Level 3	107	0	0	0	0
Total	386	54	27	30	17

**Table 2:** Some examples of mapping results between abnormal state ontology and external resources. Blank cells mean that abnormal states defined in our ontology are not existent in other resources

Concepts	Our Ontology	PATO	HPO	MeSH	SNOMED–CT
Level 1	structural abnormality				
Level 1	material degeneration	PATO:0002037 degeneration			
Level 1	hardening	PATO:0000386 hard			
Level 1	size abnormality				
Level 1	large in size	PATO:0000586 increased size			
Level 1	hyperfunction	PATO:0001625 increased functionality			
Level 1	dysfunction				
Level 1	movement abnormality			D009069 Movement Disorders	
Level 2	narrowed cross-sectional area of tube				
Level 2	hardening of wall				
Level 2	cellular tissue necrosis	PATO:0000647 necrotic		D009336 Necrosis	
Level 2	conoronary artery stenosis		HP:0005145 Coronary artery stenosis	D023921 Coronary Stenosis	233970002 Coronary artery stenosis
Level 2	arterial occlusion				2929001 Occlusion of artery
Level 2	coronary artery occlusion			D054059 Coronary Occlusion	63739005 Coronary occlusion
Level 2	chest pain		HP:0100749 Chest pain	D002637 Chest pain	29857009 Chest pain
Level 3	coronary artert stenosis in arteriosclerosis				
Level 3	esophagel stenosis in esophagitis				

Another interesting finding is that our abnormal ontology can fill the conceptual gap between abstract PATO concepts and organ-specific HPO concepts. For example, *mitral valve insufficiency* in HPO, which means an imperfect state of the closure function of the *mitral valve*, corresponds to *mitral incompetence* at Level 2 of our abnormal state ontology. On the other hand, PATO’s *insufficient* corresponds to *dysfunction* at Level 1 of our abnormal state ontology. Then, these two are connected via *valve incompetence* in our ontology. In addition, the fact that *valve incompetence* subsumes *tricuspid incompetence* demonstrates that the concepts at Level 2 of our abnormal state ontology can help find hidden subsumption relations between concepts in PATO and HPO.

#### Abnormality Ontology as Linked Data

While causal chains of abnormal states are published as linked data based on the RDF model for the causal chains of diseases, we export the *is-a* hierarchy of abnormal states in the abnormality ontology in OWL format using the OWL export function in Hozo [12] and directly publish it as linked data because it does not have particularly complicated conceptual structures.

### Development of *Disease Compass*

#### Disease Compass

To exploit the value of a disease ontology as a knowledge source for advanced medical information systems, it is important that the users can navigate the ontology easily and intuitively according to their interests. Medical experts may not find it easy to use SPARQL queries to obtain information about disease chains. Therefore, we have developed *Disease Compass* as a navigation system to explore the disease ontology. We designed the system so that users without experience with ontologies or linked data can easily explore disease knowledge and related information.

It is available at http://lodc.med-ontology.jp/ (demo movies are also available as attached files).

#### System architecture

**Fig. 11:**
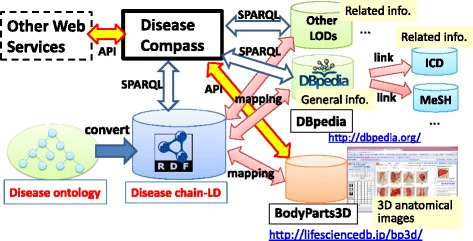
*Disease Compass* system architecture

Figure 11 shows the *Disease Compass* system architecture*.* The system obtains disease knowledge from Disease Chain-LD, which is converted from the disease ontology. It also has mapping information with other Linked Open Data (LOD) and web services, and it can obtain related information through these mappings. Although the system currently has mappings to only DBpedia and BodyPart3D, it will be possible to extend mappings to other LOD sources using existing approaches [21] to generate such linkages.

Technically, the system uses two methods to access these mapped datasets, i.e., SPARQL queries for linked data and an API for web services. If related resources (ontologies and other datasets) are published as LOD, the system can be extended easily to link such related information using SPARQL, which is the major benefit of using linked data techniques. In addition, many linked datasets include links to other data. For example, DBpedia includes links to major medical codes, such as ICD10 and MeSH; thus, the system can follow these links through mappings between Disease Chain-LD and DBpedia.

*Disease Compass* is a web service that is supported on PCs, tablets, and smartphones. It is implemented using Virtuoso as its RDF database and HTML 5 for visualization of disease chains and other information. All modules of the system provide APIs for other web services. This allows other web services to use all the functions of *Disease Compass* so that their modules will work with other related services.

#### User interface for navigation

*Disease Compass* supports the following types of navigation of the disease ontology.

1. **Navigation for definition of disease based on the RFM of a disease:** navigating definitions of diseases based on causal chains with links to other systems/datasets
2. **Navigation for general causal chains in a human body:** browsing possible causal chains of abnormal states (disorder) in the human body
3. **Navigation for definition of abnormal state (clinical disorder):** browsing the *is-a* hierarchy of abnormal state (clinical disorder) ontology with mappings to other resources

The above are related, i.e., the user can freely access other systems.

#### Navigation for definition of disease

**Fig. 12:**
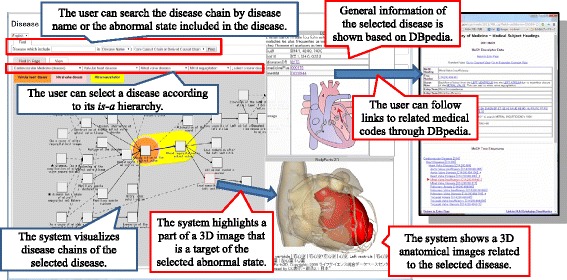
*Disease Compass* user interface

Figure 12 shows the *Disease Compass* user interface for navigating the definition of a disease. Users select a disease according to the *is-a* hierarchy of diseases, or they search a disease chain by disease name or the abnormal state included in the disease. The system visualizes the disease chains of the selected diseases in a user-friendly representation in the center of the window. The system also obtains and displays information related to the selected disease and abnormal state from the linked web services, such as general information from linked data (DBpedia) and 3D images of anatomies.

DBpedia is a linked open dataset extracted from Wikipedia [22]. We use DBpedia English (http://dbpedia.org) and Japanese (http://ja.dbpedia.org). DBpedia provides general information about diseases; however, medical experts have not approved this content. Nevertheless, we suggest that the content is sufficiently valuable to provide an overview of various diseases. In addition, DBpedia also provides links to major medical terminology and codes, such as ICD10 and MeSH, which allows users to gather specialized information about a given disease. This technology, with which related information from other web resources (e.g., ontologies, medical codes, and datasets) can be obtained through mappings, is easy to apply to other linked data. We plan to extend the target linked data in the near future.

A web service named BodyPart3D/Anatomography [23] is employed to generate 3D images of anatomies. The target area of the image is decided by *Disease Compass*, which combines all targets of abnormal states appearing in the definition (causal chains) of the selected disease chain. Subsequently, the system highlights the part of the 3D image that is the target of the selected abnormal state in the disease chain.

The functionality of *Disease Compass* is enabled because of the successful combination of our disease ontology and other web resources based on linked data technologies. As a result, *Disease Compass* allows users to explore disease knowledge and related information through various web resources.

An additional movie file shows a demonstration of navigation for definition of disease [see Additional file 1]’.


Additional file 1: A demonstration of navigation for definition of disease using *Disease Compass*. (MP4 28362 kb)


#### Navigation for general causal chains

When viewing the definition of a disease, the user selects a target abnormal state and can use the click menu to trace the causes and/or effects that form the selected abnormal state. Then, a view for navigating general causal chains is shown. In this view, users can browse the possible causal chains of abnormal states (disorders) in the human body through different diseases.

**Fig. 13:**
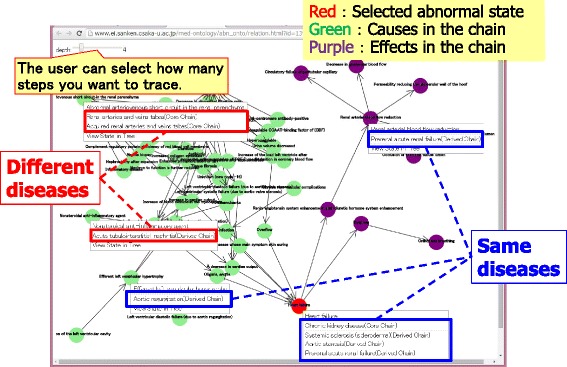
View for navigating general causal chains

Figure 13 shows an example of navigation for general causal chains whose starting point is heart failure. The red node represents the starting point, purple nodes are effects, and green nodes are causes. By right clicking, diseases that include the selected abnormal state are listed, and the system shows the definition of the selected disease when its name is clicked in the list. Note that some abnormal states shown in the view appear in different diseases when they are linked to the same chain. In particular, it is important that the user can obtain both the derived causal chains defined by the clinician directly and the causal chains derived by tracing the general causal chains through all clinical areas.

An additional movie file shows a demonstration of navigation for general causal chains [see Additional file 2]’.


Additional file 2: A demonstration of navigation for general causal chains of disease using *Disease Compass*. (MP4 45031 kb)


#### Navigation for definition of abnormal state (clinical disorder)

**Fig. 14:**
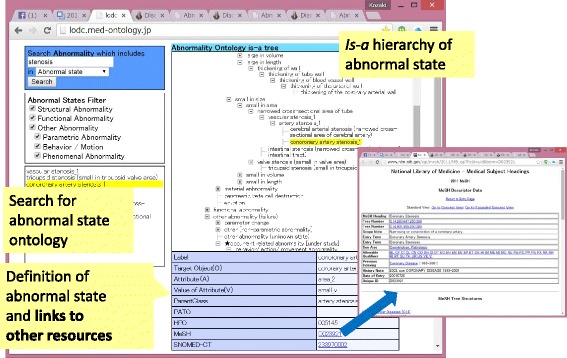
Browsing system for the abnormality ontology as linked data

As discussed in Development of *Disease Compass* section, we investigated the differences in the hierarchical structure of biomedical resources and conducted a trial integration of our abnormality ontology and related resources, such as PATO, HPO, and MeSH, based on ontological theory [9]. As a result, we developed a prototype of the abnormality ontology as linked data with a browsing system. By mapping information from other resources, users can access disease knowledge through our abnormality ontology and through other open resources (Fig. 14).

For example, ID of PATO shown in the definition of low force/decrease in force is used to jump into the Ontobee browser, which allows users to see various related information defined by PATO. Similarly, clicking a MeSH ID shown in the definition pane leads users to the corresponding terms defined in PubMed. For example, *myocardial ischemia* in the ontology of abnormal states is mapped to the corresponding MeSH term, i.e., *myocardial ischemia* (MeSH ID: D17202), from which users can retrieve all relevant information annotated by *myocardial ischemia* in NCBI.

## Discussion

The primary feature of the RFM of disease is a disease model based on the causal chains of clinical disorders. It is an appealing alternative representation of existing disease ontology, such as the DOID and the OGMS. The model captures the possibilities of clinical disorders (abnormal states) as causal chains and represents diseases by overlapping them. It can intuitively represent the causes of diseases, disease progression, and the downstream consequences of diseases to medical experts. Through these representations, the RFM of a disease can provide a broad picture of disease-associated processes in a way that fits well with the clinical understandings of diseases.

Publishing the disease ontology based on the RFM as linked data allows users to access rich knowledge/information in the disease ontology through a standard API. Furthermore, *Disease Compass* provides a well-organized graphical navigation function for the disease ontology as linked data with related web resources by mapping information.

In fact, *Disease Compass*, which allows users to navigate disease definitions with the help of abnormal states, enables users to learn whether an abnormal state is a disease cause or effect by identifying its position in the causal chain of the disease. For example, in the case of *hypertension*, users can easily find diseases that are caused by *hypertension*, including *hypertension disease* in cardiovascular medicine, and those that cause *hypertension* as a symptom. For example, the user may find that *chronic kidney disease*, in which *hypertension* appears in the upper stream of its causal chain, causes various *inflammation*, whereas, for *Liddle Syndrome, hypertension* appears in the lower stream of its causal chain as a result of *hyperactivity of the epithelial Na channel of amiloride-sensitive*. Thus, users can learn that an abnormal state can be a cause or an effect (symptom) of diseases thanks to the causal chain model of diseases in *Disease Compass*.

**Fig. 15:**
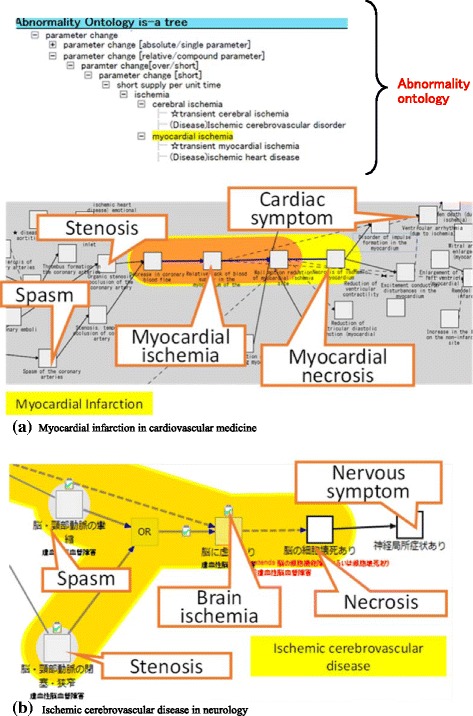
Interaction between abnormal ontology and *Disease Compass*

*Disease Compass* also allows users to compare multiple diseases to find unexpected commonalities. For example, in the case of *ischemia*, for *myocardial infarction* in cardiovascular medicine and *ischemic cerebrovascular disease* in neurology, although the locations where the corresponding abnormal states occur differ, both causal chains share a similar path up to *ischemia* (Fig. 15). In fact, both causal chains have structural disorders, such as *stenosis* and *occlusion,* or *ischemia* is caused by a *spasm* via decreased blood flow, and eventually *necrosis* occurs in either case. After *necrosis* occurs, succeeding symptoms are quite different according to *myocardial necrosis* or *necrosis of brain cells*, as shown in Fig. 15 (a)), in which symptoms (e.g., *ventricular wall motion abnormalities*) occur in the cardiovascular system, and in Fig. 15 (b), in which different symptoms (e.g., *paralysis*) are caused in locations governed by the nervous system, thereby reflecting the differences between respective organs.

**Fig. 16:**
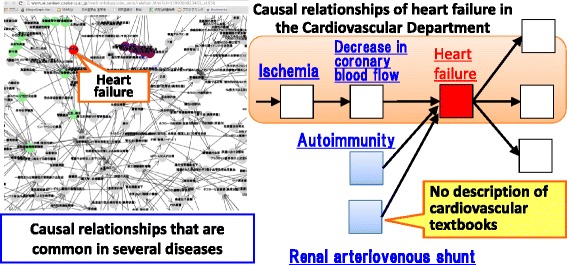
Causal relationships of heart failure

*Disease Compass* helps users uncover hidden relations between different diseases across divisions (Fig. 16). It can be done through general causal chains which include common abnormal states between different diseases across medical divisions. For example, while *heart failure* is a typical disease in cardiovascular internal medicine, *Disease Compass* can show that it can be caused by *autoimmunity* in *systemic scleroderma* in allergology and rheumatology. Another disease that causes *heart failure* in different medical fields is *renal arteriovenous fistula (aneurysmal type)* whose abnormal state, i.e., *renal arteriovenous shunt*, can also cause *heart failure*, which would be informative for novices because not all textbooks mention this rare fact.

In summary, the benefits of abnormal states organized in terms of the subsumption relation between states are significant. This helps users fill conceptual gaps between concepts in external resources and reveal hidden commonality between diseases in different medical fields. This can be realized because all diseases are described in terms of the causal chains of abnormal states and are organized in an ontology.

## Conclusions

This paper has discussed a navigation system for disease knowledge based on a disease ontology and linked data technologies. Our ontology defines diseases based on the causal chains of abnormal states (disorders), and a browsing system allows users to explore the definitions of diseases with related information obtained from linked data. We believe that this system will allow users to gain a broader understanding of diseases according to their interests and intentions.

The following list shows the summary of our contribution to clinicians and information scientists;

1. For clinicians
  They can access the basic information of diseases with causal chains through navigation using the Disease Compass. That is, they can know possible causes and/or effects of the disease of interest.
  They can find (retrieve) diseases according to causes or effects.
  They can access related resources through mapping information.
2. For information scientists
  6302 diseases and 21,699 abnormal states are defined by clinicians as a fact repository causal chains of diseases based on the RFM. It shows that the RFM was applicable to a variety of diseases.
  1554 diseases and 7080 abnormal states in six major clinical areas, which are extracted from the above RFM-diseases, are published as linked data (RDF) with SPARQL endpoint (accessible API). We call the linked data Disease Chain LD. Information scientists can access it using friendly SPARQL queries through a RDF model we designed for causal chain of diseases.
  A navigation system for disease knowledge using the Disease Chain LD, called Disease Compass, is developed. It provides navigating functions for causal chain of diseases and related information through links to other resources with GUI. It shows how the Disease Chain LD can be used for developing information systems.

The system was evaluated informally by medical experts in several meetings and workshops, and positive comments were received. A full-scale user evaluation is to be conducted in future.

Future work will also include extending the related resources using linked data and development of more practical applications using the Disease Chain-LD. The system is also subject to continuous improvement, including bug fixes and development of new functions. There remain a few topics on diseases to explore. One is a notion of an imbalance model that models pre-clinical manifestation based on the *disturbance of homeostasis* and roughly corresponds to OGMS’ disposition [1]. Another topic is identity tracking of a disease to capture its progression [24]. We must consider these significant topics and their computational models.

The latest version of *Disease Compass* is available at http://lodc.med-ontology.jp/.

## Abbreviations

DOID: Human Disease Ontology
IDO: Infectious Disease Ontology
LOD: Linked Open Data
OGMS: Ontology of General Medical Sciences
OWL: Web Ontology Language
RDF: Resource Description Framework
RFM: River Flow Model
SNOMED-CT: Systematized Nomenclature of Medicine-Clinical Terms

## Footnotes

1 Though the type 1 and type 2 diseases are not medical terms, we use these terms only in this paper to mention two kinds of diseases.
^2^We defined “small in area” as a property that an area is smaller than a given threshold. On the other hand, “narrowing tube” is defined that a cross section of a tube is smaller than a given threshold. In the same ways, definitions of “arterial/vascular stenosis” are “the cross section of an artery/blood vessel is smaller than a given threshold.” Considering these definitions, we consider that “arterial stenosis is-a vascular stenosis is-a narrowing tube is-a small in area.”
